# Whole-genome sequencing of phenotypically distinct inflammatory breast cancers reveals similar genomic alterations to non-inflammatory breast cancers

**DOI:** 10.1186/s13073-021-00879-x

**Published:** 2021-04-26

**Authors:** Xiaotong Li, Sushant Kumar, Arif Harmanci, Shantao Li, Robert R. Kitchen, Yan Zhang, Vikram B. Wali, Sangeetha M. Reddy, Wendy A. Woodward, James M. Reuben, Joel Rozowsky, Christos Hatzis, Naoto T. Ueno, Savitri Krishnamurthy, Lajos Pusztai, Mark Gerstein

**Affiliations:** 1 Program in Computational Biology and Bioinformatics, Yale University, 266 Whitney Ave., Bass 432A, New Haven, CT 06520 USA; 2 Yale Cancer Center, Breast Medical Oncology, Yale School of Medicine, 300 George Street, Suite 120, Rm133, New Haven, CT 06511 USA; 3 Department of Molecular Biophysics and Biochemistry, Yale University, 266 Whitney Ave., Bass 432A, New Haven, CT 06520 USA; 4 Center for Precision Health, School of Biomedical Informatics, University of Texas Health Science Center Houston, Houston, TX USA; 5 Department of Medicine, Massachusetts General Hospital, Harvard Medical School, Boston, MA USA; 6 Department of Biomedical Informatics, College of Medicine, The Ohio State University, Columbus, OH USA; 7 The Ohio State University Comprehensive Cancer Center (OSUCCC – James), Columbus, OH USA; 8 Division of Hematology/Oncology, Department of Internal Medicine, University of Texas Southwestern Medical Center, Dallas, TX USA; 9 Department of Breast Medical Oncology, The University of Texas MD Anderson Cancer Center, Houston, TX USA; 10 Morgan Welch Inflammatory Breast Cancer Research Program and Clinic, The University of Texas MD Anderson Cancer Center, Houston, TX USA; 11 Department of Radiation Oncology, The University of Texas MD Anderson Cancer Center, Houston, TX USA; 12 Department of Hematopathology, The University of Texas MD Anderson Cancer Center, Houston, TX USA; 13 Department of Computer Science, Yale University, 266 Whitney Ave., Bass 432A, New Haven, CT 06520 USA; 14 Department of Statistics and Data Science, Yale University, 266 Whitney Ave., Bass 432A, New Haven, CT 06520 USA

**Keywords:** Inflammatory breast cancer, Whole-genome sequencing, Single nucleotide variant, Copy number variant, Structural variant

## Abstract

**Background:**

Inflammatory breast cancer (IBC) has a highly invasive and metastatic phenotype. However, little is known about its genetic drivers. To address this, we report the largest cohort of whole-genome sequencing (WGS) of IBC cases.

**Methods:**

We performed WGS of 20 IBC samples and paired normal blood DNA to identify genomic alterations. For comparison, we used 23 matched non-IBC samples from the Cancer Genome Atlas Program (TCGA). We also validated our findings using WGS data from the International Cancer Genome Consortium (ICGC) and the Pan-Cancer Analysis of Whole Genomes (PCAWG) Consortium. We examined a wide selection of genomic features to search for differences between IBC and conventional breast cancer. These include (i) somatic and germline single-nucleotide variants (SNVs), in both coding and non-coding regions; (ii) the mutational signature and the clonal architecture derived from these SNVs; (iii) copy number and structural variants (CNVs and SVs); and (iv) non-human sequence in the tumors (i.e., exogenous sequences of bacterial origin).

**Results:**

Overall, IBC has similar genomic characteristics to non-IBC, including specific alterations, overall mutational load and signature, and tumor heterogeneity. In particular, we observed similar mutation frequencies between IBC and non-IBC, for each gene and most cancer-related pathways. Moreover, we found no exogenous sequences of infectious agents specific to IBC samples. Even though we could not find any strongly statistically distinguishing genomic features between the two groups, we did find some suggestive differences in IBC: (i) The *MAST2* gene was more frequently mutated (20% IBC vs. 0% non-IBC). (ii) The TGF *β* pathway was more frequently disrupted by germline SNVs (50% vs. 13%). (iii) Different copy number profiles were observed in several genomic regions harboring cancer genes. (iv) Complex SVs were more frequent. (v) The clonal architecture was simpler, suggesting more homogenous tumor-evolutionary lineages.

**Conclusions:**

Whole-genome sequencing of IBC manifests a similar genomic architecture to non-IBC. We found no unique genomic alterations shared in just IBCs; however, subtle genomic differences were observed including germline alterations in TGFβ pathway genes and somatic mutations in the *MAST2* kinase that could represent potential therapeutic targets.

**Supplementary Information:**

The online version contains supplementary material available at 10.1186/s13073-021-00879-x.

## Background

Inflammatory breast cancer (IBC) is a rare form of breast cancer with very little known about its molecular etiology that is responsible for its aggressive clinical course. IBC accounts for 2–4% of all breast cancers in the USA [1] and causes 7–10% of breast cancer-related deaths in Western countries [2, 3]. IBC includes all known molecular subtypes of breast cancer, but they are considerably more aggressive than in non-IBC, with poorer disease-free survival and overall survival [2, 4]. The disease often presents with rapidly progressing symptoms of swelling of the breast, redness, and thickening of the skin of the breast which resembles an active inflammatory process, which led to the name of the disease. However, the symptoms are not caused by inflammatory cells, but by cancer cells blocking lymph vessels in the skin and breast parenchyma [5]. IBC also has a propensity for rapid dissemination and distant metastatic spread. Gene expression profiling studies have not revealed any consistent IBC-specific gene expression patterns; consequently, there is no molecular diagnostic test to define this disease [4, 6]. The diagnosis is based on the unique and rapidly progressive clinical features of the cancer. Targeted sequencing of ~ 200 cancer-related genes in IBC showed that the most frequently altered gene was TP53, with reported frequencies between 43 and 75% [7–9]. Currently, there is no whole-exome or whole-genome sequence data available for IBC and its DNA level alterations have not been characterized. We hypothesize that specific DNA sequence changes in the coding or non-coding regions of the genome may be responsible for the unique phenotype of IBC. The goal of this project was to perform deep characterization of the complete genomic features of IBC specimens to identify IBC-specific sequence alterations that could potentially explain its etiology and provide new diagnostic markers.

## Methods

### Tissues

Twenty IBC tissues and paired normal DNA from blood were obtained from the Morgan Welch Inflammatory Breast Cancer Research Program and Clinic at MD Anderson Cancer Center under an IRB-approved study. All IBC tissues were individually reviewed by a breast pathologist (Savitri K.) and a clinical investigator (N.U) for accuracy of diagnosis and to ensure tumor cellularity > 60%. All patients provided informed consent for genomic analysis of their cancer and germline DNA. Characteristics for 20 IBC patients are shown in Additional file 1: Table S1. Twenty-three non-IBC samples were selected from the Cancer Genome Atlas (TCGA) study of breast cancer cohort that were proportionally matched by molecular subtype, clinical stage, age, and race. This was done to ensure that various covariate distributions were similar between the IBC and the non-IBC samples studied in this project. Characteristics for those selected non-IBC samples are shown in Additional file 1: Table S1.

### DNA extraction

DNA was extracted from the snap frozen core needle biopsy of the breast tumor and peripheral blood using the QiAamp DNA Mini kit (Qiagen). The tissue was disrupted in buffer ATL, homogenized, and then lysed using Proteinase K. Buffer AL and ethanol was then added to the lysate creating conditions that promoted selective binding of the DNA to the QIAamp spin columns. The sample was then applied to the mini spin columns. The DNA bound to the membrane was eluted in buffer TE at pH 8.0.

### Whole-genome sequencing

One-microgram germline and tumor DNA were used for WGS that was performed under a Yale IRB-approved protocol (*HIC #1406014226*). It was performed on the Illumina HiSeq 2500 sequencing platform at Macrogen. The samples were prepared according to the Illumina TruSeq DNA library preparation guide. The 150-base pair (bp) paired-end libraries were sequenced with median coverage of 60X for the tumor samples and 40X for the matched normal samples. Detailed sequencing information, including sequencing depth, raw and mapped read numbers, and mapping rates, is summarized in Additional file 2: Table S2.

### Sequence alignment and qualify control

We mapped raw FASTQ files for tumor and matched normal samples for 20 IBC samples to the hg19 reference genome using BWA-MEM [10] algorithm with default parameters. Subsequently, reads were sorted and duplicate reads were marked using Samtools [11] and Picard tools (http://broadinstitute.github.io/picard) to obtain the final set of BAM files for variant calling. The BAM files have been deposited in the European Genome-Phenome Archive (EGA) under EGA accession EGAS00001004117 (https://wwwdev.ebi.ac.uk/ega/studies/EGAS00001004117). For the non-IBC samples, we followed the same procedure. Details of sequencing for each sample are summarized in Additional file 2: Table S2.

### Germline SNV and INDELs calling

For both IBC and non-IBC cohorts from TCGA, we generated the germline SNVs and INDEL call set using the GATK tool [12]. Briefly, we followed the GATK best practice to call germline variants. We realigned the original bam using IndelRealigner and base recalibrator module in the GTAK. Subsequently, variants were called using GATK HaplotypeCaller algorithm. Raw variants were filtered using the variant recalibration module in the GATK. Briefly, the variant recalibration method uses a continuous adaptive error model, while taking into account of the relationship between variant and the probability of it being a true positive instead of a sequencing artifact.

### Somatic SNV and INDELs calling

We called somatic variants for IBC and non-IBC samples from TCGA using MuTect [13] and Strelka [14] tools. Briefly, these tools take tumor and matched normal bam files as input to identify somatic variants supported by minimum number of reads. Somatic SNVs in this study were based on MuTect and Strelka, whereas somatic INDELs were called using Strelka. The initial PASS only call set obtained from both MuTect and Strelka were further filtered for potential germline contaminated call by removing common variants as defined in the 1000 Genomes Project [15]. Furthermore, we also removed somatic SNVs and INDELs falling outside the high mappability regions of the genome as defined by the Genome in a Bottle Consortium (GIAB) [16]. Finally, we took the intersection of MuTect and Strelka call sets and removed those somatic SNVs and INDELs that appeared in germline call set.

### Detection of loss of heterozygosity (LOH) in tumor DNA

For each site of germline SNV identified from a normal blood sample, we determined the corresponding somatic genotype in tumor DNA by using Samtools [11] and Bcftools [17], which reported information for reference allele, alternative allele, allele count, and allele frequency. An LOH event was identified when the site met these two criteria: (i) it was called as a heterozygous variant (alternative allele frequency = 0.5) in normal blood DNA and (ii) it was shown as homozygous (alternative allele frequency = 0 or 1) in tumor DNA.

### Somatic SV calling

We applied Meerkat [18] to identify somatic structural variants in the IBC and the non-IBC cohorts from TCGA. Briefly, Meerkat extract soft-clipped and unmapped reads from the bam files. These reads are subsequently remapped to the reference genome using BLAT [19] to identify discordant read pairs for SV discovery. Meerkat also characterizes breakpoint around the SVs to assign the underlying mechanism generating SVs. Meerkat generated SVs were further filtered based on mappability criterion and supporting read pairs > 2.

### Identification of somatic CNVs

We implemented BIC-Seq2 [20] to call somatic CNVs using default parameters. In the SeqNorm step, we set read length to 151 bp and bin size to 1000 bp. The fragment size was calculated using the first one million properly mapped reads with mapping quality at least 20 in the BAM files.

We also used a signal processing approach for filtering the somatic copy number segments (sCNSs) identified by BIC-Seq2 in the last step. In this analysis, we focused on large-scale events affecting > 100 kB in length. Below are the specific procedures:

1. For each sample, compute the read depth (RD) signal levels using the mapped reads. This is done by counting the number of reads that overlap with each base. For each patient, we computed RD signal for tumor and the matching normal tissue.
2. Next we normalized normal tissue profiles using reads per million normalization. Given *i*^th^ sample’s tumor and normal signal profiles, we multiplied the normal signal profile with the ratio of total RD signal in tumor and total RD signal in normal.
  $$ \hat{R{D}_n}(i)=R{D}_n(i)\times \frac{\sum R{D}_t(j)}{\sum R{D}_n(j)} $$

$\hat{R{D}_n}(i)$ denotes the normalized normal RD signal at *i*^th^ base position in the genome.

3. We next divided the genome into 3000 bp bins and computed the total tumor signal and normal sample’s normalized RD signal in each bin.
4. We next computed the log ratio (LR) profiles by dividing the total tumor RD signal by normal sample RD signal in each bin and computing the log_2_ of this ratio. This profile represents a measure of the deletions (LR < 0) and amplifications (LR > 0)
  $$ \mathrm{LR}(b)={\log}_2\frac{\sum_iR{D}_t(i)}{\sum_{i\in {c}_b}\hat{R{D}_n}(i)} $$

where *c*_*b*_ = [(*b* − 1) ∙ *l*_*bin*_, *b* ∙ *l*_*bin*_] represents the base positions for *b*^*th*^ bin.

5. The LR profile is generally extremely noisy. We use median-based smoothing to smooth the signal. We use a sliding window approach where window size is set to 1000 bins and replace the LR value at each bin with the median of the LR values within the 1000 bins’ vicinity. The smoothing operation removes substantial amount of noise from the LR signal
  $$ \mathrm{LR}(b)=\mathrm{median}\left(\mathrm{LR}\left(b-{l}_{win}\right),\dots, \mathrm{LR}\left(b+{l}_{\mathrm{win}}\right)\right) $$
6. Next, we identify sCNS by evaluating the regions where smoothed LR is constant. On each sCNS, we assign the tumor-to-normal log ratio signal by computing the ratio of total tumor to total normal RD signal. The segments with LR < 0 are assigned as deletions and segments with LR > 0 amplifications. We denote the LR value for segment *s* on sample *k* with $L{R}_s^k$
  Finally, we only took the strongest calls from BIC-Seq2, after filtering by the signal processing approach introduced above, as the final call set. Copy number gain was defined as log2 (tumor/expected) ratio > 0.2. Copy number loss was defined as log2 (tumor/expected) ratio < − 0.2.

### Functional annotation and impact prediction

Both somatic and germline SNVs were annotated by FunSeq2 [21]. Since non-coding variants in regulatory elements (promoter, enhancer, etc.) can be associated with potential target genes, this pipeline helps to identify both coding and non-coding variants of a given gene. Additionally, functional impact of each variant was predicted by PredictSNP2 [22], which could be neutral, deleterious, or unknown. Only deleterious (high functional impact) variants were selected for gene and pathway-level analysis.

### Identification of candidate driver genes

Candidate driver genes in IBC cohort were detected by ActiveDriverWGS [23] with default parameters. Final call sets for all somatic SNVs in IBC samples were used as input. Coordinates of genes were extracted from Ensemble database under hg19 reference genome, using biomaRt package [24]. Genes with FDR < 0.05 were identified as candidate drivers.

### Mutation spectra and mutational signatures

Somatic SNVs across the whole genome were analyzed in single nucleotide and tri-nucleotide context, respectively. DeconstructSig [25] was used to deconstruct the mutation spectrum (96 possible tri-nucleotide combinations) of each sample into 30 reference mutational signatures in the COSMIC database [26], in order to calculate the weight of each reference signature.

### Estimation of number of clones

We implemented SciClone [27] to estimate the number of clones for each IBC and non-IBC sample. First, all somatic SNVs with allele frequency higher than 0.6 were removed from the input file as they were likely affected by copy number loss events. Next, function “sciClone” was called with “minimumDepth” set as 14 and “clusterMethod” set as “binomial.bmm”. Finally, the output of the function reported the predicted number of clones detected in the given sample. After repeating the above procedures for all IBC and non-IBC samples, the predicted numbers of clones were compared between two cohorts by Fisher’s exact test.

### Evolutionary trees build-up using PhyloWGS

We used PhylowWGS [28] to infer the evolutionary trees for each individual sample. We followed a similar workflow as previously described [29]. Somatic SNVs from the consensus calls of Strelka and Mutect were used. The observed alternative allele and reference allele counts were from Strelka. To remove copy number effects, we removed SNVs in the regions with an absolute “log2.copyRatio” (log2 tumor to normal copy number ratio, reported by BIC-Seq2) higher than 0.2 and *p*-value lower than 0.01. Then, we ran PhyloWGS [28] using default parameters and set genders all to female. We only plotted and analyzed the tree with the highest likelihood reported by PhyloWGS.

### Estimation of tumor purity

In order to estimate the tumor purity for each IBC tumor sample, a computational pipeline called PurBayes [30] was implemented with default parameters. Estimation results were summarized in Additional file 3: Table S3.

### Microorganism sequences’ detection and enrichment

In order to identify sequences that are potentially of exogenous origin and not arising of the host genome, we modified a portion of the exceRpt pipeline that was developed for the identification of endogenous and exogenous extracellular RNAs [31]. After reads are aligned to the host genome, we performed a second pass alignment against the host genome in order to remove sequences that might potentially come from the host human genome. We then removed reads that align with a high number of mismatches (5 mismatches per 100 bp). We also filter out reads that align against repetitive sequences in the human genome and reads that multi-map up 200 locations in the human genome. While we cannot confidently assign these reads to the human genome, the goal is to filter them out in order to obtain a set of reads that we are confident that do not come from the host human genome.

These reads are then aligned against indices for a set of full genomes for all sequenced bacteria, viruses, plants, fungi, protist, metazoa, and the following 12 vertebrate genomes: chicken, cod, cow, dog, duck, frog, horse, rabbit, pig, sheep, tilapia, and turkey. Since many exogenous genomes have a high degree of sequence similarity based on evolution, we find that many reads that align to an exogenous genome align to multiple genomes. By default, the pipeline allows for no mismatches during this step (in order to be as conservative as possible in identifying possible exogenous sequences). We assign reads that align to exogenous genomes to the position in the phylogenetic taxonomy tree based on the node that is most parsimonious with the different genomes that the read aligns.

### Validation cohorts

In order to validate key genomic findings identified from IBC cohort, we expanded our analysis to multiple other cohorts, including breast cancer and other types of cancers, as well as general population. More specifically, we investigated high-functional impact mutation frequencies of genes and pathways in (1) PCAWG breast cancer cohort [32], (2) twenty-three types of primary cancers from ICGC (https://dcc.icgc.org/), and (3) general population data from The Genome Aggregation Database [33].

### Statistical analysis

There are two types of statistical testing methods used in this study: (1) Wilcoxon rank-sum test and (2) Fisher’s exact test. Wilcoxon test was implemented when comparing median of IBC and non-IBC samples. Fisher exact test was used when comparing fraction of IBC and non-IBC samples in each category. All original *p*-values from above tests were adjusted by the very conservative Bonferroni correction. Adjusted *p*-values< 0.05 were considered statistically significant. Additionally, we implemented randomization test to validate the statistical significances by three steps: (1) mix IBC and non-IBC samples and randomly assign them to two groups, (2) test the significance under new sample labeling, and (3) Repeat the analysis for 1000 times and summarize the statistics. All statistical analysis was performed by R software (https://www.r-project.org/). R packages ggplot2 (http://ggplot2.org), ComplexHeatmap [34], and RCircos [35] were used to visualize the results.

## Results

### Somatic mutation burden and functional annotations

**Fig. 1:**
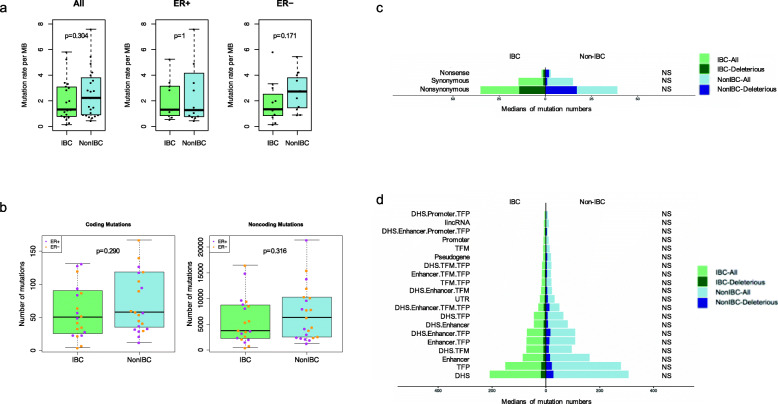
Mutation burden and functional annotations. **a** Rates of somatic SNVs in IBC and non-IBC cohorts, for all samples, and for ER+ and ER− cases separately. *P*-values are from Wilcoxon rank sum test. **b** Number of coding and noncoding somatic SNVs. Each dot represents a sample color-coded by ER status. *P*-values are from Wilcoxon rank sum test. **c** Medians of somatic SNVs for various types of coding mutations. **d** Median numbers of noncoding SNVs by functional class in IBC and non-IBC. Light and dark bars on panels **c** and **d** correspond to the numbers for all mutations and deleterious mutations, respectively. Number of mutations in each annotation category was compared between two cohorts by Wilcoxon rank sum test, resulting in *p*-values ranging from 0.14 to 0.63. Similar tests were implemented for deleterious variants only for each annotation category, with *p*-values 0.10–0.93. Fractions of deleterious mutations were tested by two-proportions *z*-test with Yates’ continuity correction, showing all *p*-values were > 0.05 for each unique annotation category. “NS” in panels **c** and **d** represent that all *p*-values are not significant (*p* > 0.05)

WGS identified 114,563 somatic SNVs in 20 IBC samples (range 424–16,662 per tumor; median 3789), among which 1282 variants (1.12%) were in coding regions. IBC and non-IBC showed similar mutation rate per megabase (MB) (Fig. 1a). The number of somatic coding and noncoding SNVs were similar between the IBC and non-IBC cohorts (Fig. 1b). Noncoding somatic SNVs were annotated with FunSeq2 [21] into 20 different, non-overlapping functional categories. The number of somatic SNVs within each annotation category was similar between the two cohorts (Fig. 1c, d).

### Mutation spectra and mutational signatures

IBC has similar proportions of base changes as non-IBC, for all single-nucleotide mutation contexts (C>A, C>G, C>T, T>A, T>C and T>G) (Additional file 4: Fig. S1a) (Wilcoxon test, adjusted *p*-values> 0.05 by Bonferroni method), as well as tri-nucleotide mutation contexts (Additional file 4: Fig. S1b) (Wilcoxon test, adjusted *p*-values > 0.05 by Bonferroni method). The mutation spectrum of each sample was deconstructed using DeconstructSig [25] into 30 reference mutational signatures in the COSMIC database [26]. IBC and non-IBC samples showed no difference in mutational signature distribution (Additional file 4: Fig. S1c) (Wilcoxon test, adjusted *p*-values> 0.05 by Bonferroni method). In particular, there was no difference in signature 3, which has been associated with homologous recombination defect (HRD) in breast cancer [36]. Statistical comparison of weights of signature 3 indicates that IBC samples have similar degree of HRD to non-IBC ones (Additional file 4: Fig. S1d) (Wilcoxon test, *p* = 0.85).

### Copy number variants and structural variants

**Fig. 2:**
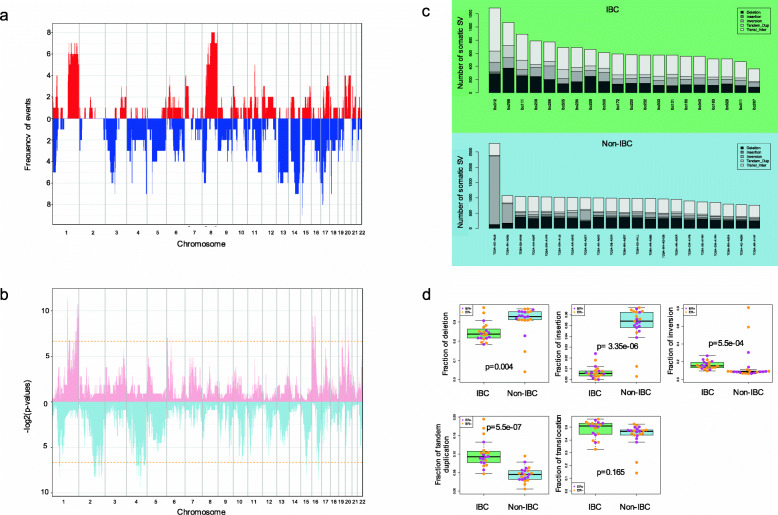
Somatic copy number variants and structural variants. **a** Somatic copy number profile of the IBC cohort. *X*-axis represents genome coordinates ordered by chromosomes. *Y*-axis shows the frequency of copy number gain (red) and copy number loss (blue) in 1 Mb-length bins across the genome in IBCs. **b** Significance of differences of copy number profiles between IBC and non-IBC cohorts. *X*-axis shows genome coordinates by chromosome and the *Y*-axis shows the log-transformed *p*-value from the Fisher’s exact test, obtained from the comparison of frequencies of copy number gain (pink) and copy number loss (light blue) events between two cohorts. Dashed lines represent *p*-value = 0.01. All significant peaks (Bonferroni-adjusted *p*-value < 0.01) have less frequency in IBC, for both copy number loss and gain events. **c** Number of somatic SVs in individual IBC and non-IBC samples. Shades represent the types of somatic SVs. **d** Fractions of each type of somatic SVs in IBC and non-IBC cohorts. Each dot represents a sample color-coded by its ER status. *P*-values were calculated by Wilcoxon test and adjusted by Bonferroni method

Copy number loss or gain events were mapped into 1-MB-sized bins across the entire genome (Fig. 2a, Additional file 4: Fig. S2). For each bin, frequencies of copy number loss or gain events were summarized separately and then compared between IBC and non-IBC cohorts. For copy number gain events (defined as log2 (observed tumor/expected) ratio > 0.2), 108 peaks showed significantly different frequencies between two cohorts, locating at chromosome 1, 3, 6, 16, 17, 19, and 20 (Additional file 5: Table S4) (Fisher’s exact test, *p*-values< 0.05), in contrast to 34 significant peaks reported by the randomization test (median, 34; minimum, 16; maximum, 62). On the other hand, for copy number loss events (defined as log2 (observed tumor/expected) ratio < − 0.2), 221 peaks showed significantly different frequencies between two cohorts, locating at chromosome 1, 2, 4, 5, 9, 10, 11, 12, 15, 16, and 17 (Additional file 5: Table S4), in contrast to 57 significant peaks reported by the randomization test (median: 57; minimum: 38; maximum: 80). There were 26 cancer-related genes involved in these differentially affected genomic regions (Additional file 6: Table S5), including *LRP1B* as a putative tumor suppressor gene, and *ERBB4* as a member in the EGFR subfamily of receptor tyrosine kinases.

Large structural variants were classified into five categories: deletion, insertion, inversion, tandem duplication, and inter-chromosomal translocation (Fig. 2c). The fraction of large somatic SVs in each category was compared between IBC and non-IBC cohorts. IBC showed significantly higher fraction of complex events than non-IBC, including tandem duplications (median 0.093 vs. 0.045) (Wilcoxon test, Bonferroni adjusted *p* = 5.5e−07) and inversions (median 0.154 vs. 0.088) (Wilcoxon test, Bonferroni adjusted *p* = 5.5e−04) (Fig. 2d). On the contrary, IBC showed significantly lower fraction of large deletions (Wilcoxon test, Bonferroni adjusted *p* = 0.004) and insertions (Wilcoxon test, *p* = 3.4e−06), compared with non-IBC samples. Additionally, comparison of the absolute numbers of somatic SVs in each category also presented significant differences (Additional file 4: Fig. S3). For small insertions and deletions (INDELs), both categories of mutations reported similar numbers between IBC and non-IBC cohorts (Additional file 4: Fig. S4). Circos plots summarizing the combined germline and somatic genetic aberrations detected from WGS for each individual IBC are shown in Additional file 4: Fig. S5.

### High functional impact mutations and affected genes

**Fig. 3:**
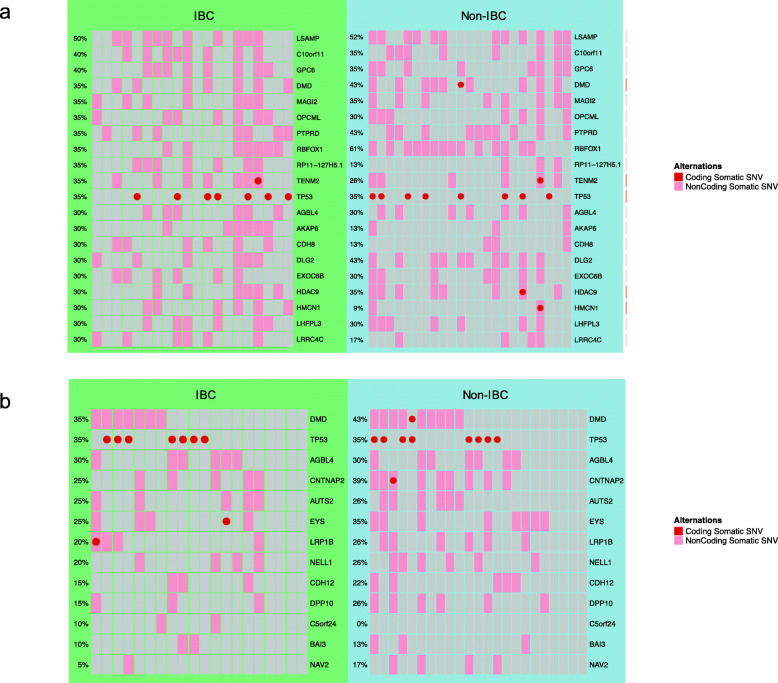
Affected genes by deleterious somatic SNVs. **a** The top 20 most frequently affected genes in the IBC cohort. **b** Candidate driver genes identified by ActiveDriverWGS (FDR < 0.05). Mutations in both coding and non-coding regions of a gene are shown. Each column represents one case (IBC or Non-IBC). Each row shows one gene. All genes in panel **a** and **b** had similar mutation frequencies in IBC and non-IBC cohorts (Fisher’s test, Bonferroni-adjusted *p*-values> 0.05)

High-functional impact (HFI) somatic SNVs were selected based on the deleteriousness predictions reported by PredictSNP2 [22], including both coding and noncoding SNVs. Affected genes were then extracted for each sample, and their mutation frequencies were compared between two cohorts. Our analysis showed that all genes with at least one high-functional impact somatic SNVs in the IBC cohort were similarly affected in the IBC and non-IBC cohorts (Fisher’s test, Bonferroni adjusted *p*-values> 0.05). For IBC, the top 20 genes most frequently affected by deleterious somatic SNVs included *LSAMP*, *GPC6*, and *TP53* among others (Fig. 3a). The top 20 most frequently affected genes by coding and non-coding deleterious somatic SNVs were summarized in Additional file 4: Fig. S6a and Additional file 4: Fig. S6b, respectively. Additionally, thirteen candidate driver genes were detected with ActiveDriverWGS in the IBC cohort (FDR < 0.05) (Fig. 3b). However, all of them showed similar mutation frequencies between the IBC and the non-IBC cohorts (Fisher’s test, Bonferroni adjusted *p*-values> 0.05) (Fig. 3b).

Notably, we identified four of 20 (20%) IBCs had unique predicted deleterious mutations in the non-coding (promoter and intron) region of *MAST2* (Microtubule-Associated Serine/Threonine-Protein Kinase 2), while no deleterious mutation was detected in any of the 23 non-IBC cases in our cohort. In the PCAWG [32] breast cancer cohort, we could find only 1 out of 198 samples (0.5%) having a mutation in this gene. In the largest WGS study for breast cancer (BRCA-EU from The ICGC Breast Cancer Project), the mutation frequency of *MAST2* was 1/569 (0.18%) [37], which was significantly lower than our IBC cohort (Fisher’s test, Bonferroni adjusted *p* = 0.024). We also determined the frequency of high-functional impact mutations in the *MAST2* gene in the ICGC Data Portal (https://dcc.icgc.org/), for 22 different primary cancer sites. We found that the two highest mutation frequencies were in thyroid cancer (3/50 = 6%) and nasopharyngeal cancer (1/21 = 4.76%), while the frequencies were < 2% in all other cancer types.

### Alterations of cancer-related signaling pathways

**Fig. 4:**
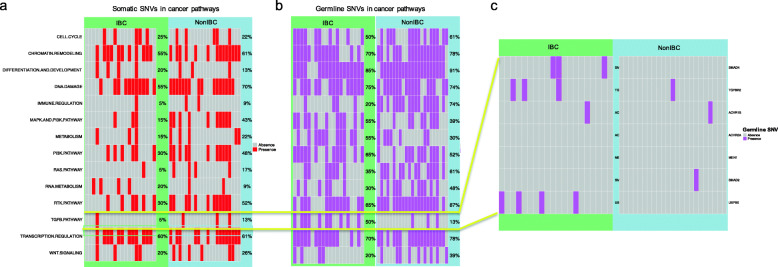
Deleterious mutations in cancer pathways. **a** Deleterious somatic SNVs in cancer pathways in IBC and non-IBC. **b** Deleterious germline SNVs in cancer pathways in IBC and non-IBC. **c** Deleterious germline SNVs in the TGF *β* signaling pathway in IBC and non-IBC. In **a** and **b**, each column represents one case (IBC or Non-IBC). Each row shows a given cancer pathway. In **c**, each column represents one case (IBC or Non-IBC). Each row shows a gene

We investigated pathway-level aberrations in 14 cancer-related biological pathways [38]. For somatic SNVs, none of these pathways had significantly different mutation frequencies between the IBC and non-IBC cohorts (Fisher’s test, Bonferroni adjusted *p* > 0.05) (Fig. 4 (a)). For germline SNVs, the IBC cohort showed a significantly lower frequency of aberrations in the immune regulation pathway than non-IBC (Fisher’s test, Bonferroni adjusted *p* = 0.009) (Fig. 4 (b)). Randomization test (*N* = 10,000) showed that the probability of observing a significant difference in the immune regulation pathway was 21/10,000 = 0.0021, which is significant.

Previous studies have identified the TGF *β* pathway as a potential therapeutic target in IBC [39, 40]. In this study, we observed a numerically higher (but not statistically significant after correction for multiple testing) mutation frequency of predicted deleterious germline SNVs in the TGF *β* pathway in IBCs (50% vs. 13%, Fisher’s test, Bonferroni adjusted *p* = 0.25) (Fig. 4 (b)). Seven IBC cases (35%) had deleterious germline SNVs in either *SMAD4* or *USP9X*, both involved in TGFβ signaling; one of these cases had variants in both the coding and non-coding regions and the rest of cases only had non-coding variants (Additional file 7: Table S6). However, none of the non-IBC cases had any deleterious germline SNVs in these two genes in the coding or non-coding regions (Fig. 4 (c)). We did not observe any LOH event at the corresponding genomic locations in the tumor DNA (Additional file 7: Table S6). Notably, none of these deleterious germline SNVs was detected in the ICGC breast cancer cohort (*n* = 1970), and they are also very rare in the general population, with variant allele frequency (VAF) < 0.007 in The Genome Aggregation Database (*n* = 141,456) [33] (Additional file 7: Table S6).

### Clonal architecture and evolutionary trees

**Fig. 5:**
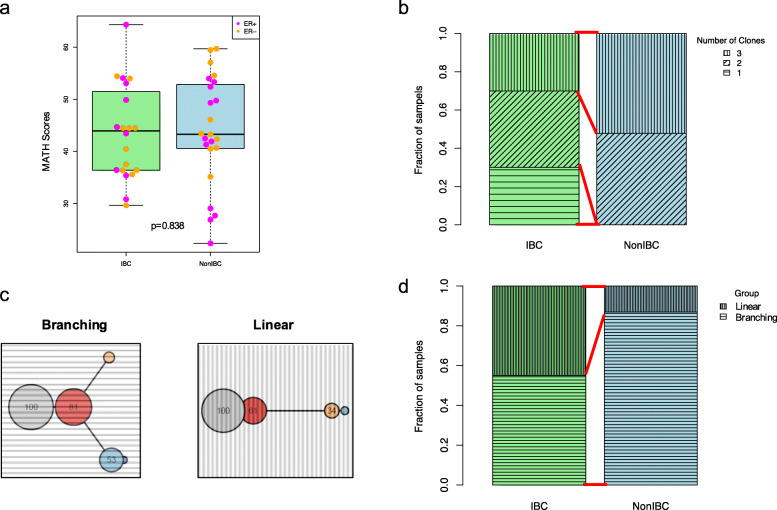
Clonal architecture and evolutionary trees. **a** MATH scores of IBC and non-IBC. Each dot represents a sample color-coded by its ER status. **b** Fraction of samples with one, two, and three clones, in IBC and non-IBC cohorts. **c** Two individual evolutionary trees showing branching and linear patterns (results for all samples are shown on Additional file 4: Fig. S4). **d** Fraction of samples classified into branching and linear groups, respectively

IBC had similar mutant-allele tumor heterogeneity (MATH) [41] as non-IBC cases (Fig. 5a). For each sample, the number of clones was estimated by SciClone [27], based on the model fitting procedures on the distribution of variant allele frequencies (Additional file 4: Fig. S7 and Additional file 4: Fig. S8). The results revealed that 6/20 (30%) IBC cancers were clonal (consisting of only one clone), whereas all non-IBC cases had at least two clones (Fisher’s test, *p* = 0.006) (Fig. 5b). We then constructed evolutionary trees for each case to further explore the mutational process heterogeneity as previously described [29] (Additional file 4: Fig. S9). These trees were derived from the whole-genome mutation calls, with their topology suggesting a temporal ordering to the mutations. We could classify the trees into two groups based on their topology: branching or linear (Fig. 5c). Nine out of 20 (45%) of IBC cancers were linear, which is significantly more than non-IBC cases (3/23, 13%) (Fisher’s test, *p* = 0.039) (Fig. 5d and Additional file 4: Fig. S9). Our results illustrated that IBC is evolutionarily more homogeneous than non-IBC, with less clonality and less complex evolutionary features. These findings may result from the faster growth of IBC tumors, compared with non-IBC ones.

### Microorganism sequences’ detection and enrichment

As IBC clinically mimics bacterial infection of the breast and/or skin [42], therefore, we also looked for infectious agents in the IBC tumor tissues. We applied a modified exceRpt pipeline [31] to examine the sequence reads that did not map to the human reference genome, in order to detect microorganism sequences in the DNA of IBC and matching normal samples. The top 100 most frequent microorganism sequences in all samples including cancer and normal were highly enriched in *Propionibacterium acnes* (Additional file 4: Fig. S10). *P. acnes* is a ubiquitous skin bacterium that represents a common source of contamination in sequencing studies that can originate from the patient or acquired during tissue handling [43]. We found no infectious agent DNA specific to the IBC cancer samples, which diminished the possibility that IBC was caused by bacterial infection.

## Discussion

Our study is the first WGS analysis of IBC. We could not identify a single genomic abnormality that is shared by all samples and therefore could molecularly define IBC. IBC tissues showed similar mutation load, mutational spectra, and mutation signatures as non-IBC, and most somatic mutations occurred at similar frequencies in both cohorts. We did not detect any cancer-specific infectious agents in the DNA extracted from IBC tissues. However, we did identify several subtle genomic differences that distinguished IBC from non-IBC in our cohort. The non-coding region of the *MAST2* gene was mutated at a higher frequency than what reported in any previous WGS analysis of breast cancer. In our IBC cohort, 20% of the cases had a mutation, while the mutation frequency of this gene in coding or non-coding regulatory regions is between 0.18 and 0.5% in non-IBC cases in the PCAWG and ICGC breast cancer WGS datasets. *MAST2* is a microtubule-associated serine/threonine kinase that interacts with the Protocadherin-LKC, a recently proposed tumor suppressor gene for colon and liver cancers, which mediates contact inhibition of cell proliferation [44]. *MAST2* also regulates lipopolysaccharide-induced IL-12 synthesis in macrophages by forming a complex with TRAF6 and inhibiting NF-kappa-B activation [45]. *MAST2* gene rearrangements were previously noted in some breast cancers, and overexpression of *MAST2* (or MAST1) gene fusions in breast epithelial cells led to increased proliferation in vitro *and* in vivo [46]. In our study, we found deleterious mutations in the non-coding regions of *MAST2*; however, the functional impact of these variants has not yet been investigated. Since *MAST2* has not been included in any of the previous targeted sequencing studies of IBC, future datasets of IBC will be needed to validate this finding.

Complex structural variants also appeared to be more common in IBC, including tandem duplication and inversion, suggesting greater genome complexity than in non-IBC. Several genomic regions showed significantly different copy number profiles harboring genes involved in cancer biology (Additional file 6: Table S5). However, sequencing platforms with different coverages and depths could introduce bias when calling large structural variants. As our IBC and non-IBC cohorts were sequenced separately, some observed differences may arise from the different sources of sequencing data.

A surprising finding of our study was the low clonality of IBC at the time of diagnosis. A substantial minority of IBC had only one detectable clone whereas all non-IBC cases had more than one clones. When we examined the evolutionary trees of the tumor cell populations, we observed two distinct groups that we describe as branching and linear evolution. IBCs showed significantly more of the linear evolutionary pattern than non-IBC (45% vs. 13%, *p* = 0.039). These results suggest that IBC cells are evolutionarily more homogeneous and exhibit lower clonality than non-IBC cancer cells, leading to the hypothesis that a high proliferation rate and rapid expansion of a single aggressive clone could be responsible for the rapid initial clinical course of the disease, which often unfolds in a few weeks. In comparison, non-IBC often grow for years before becoming detectable, which may allow for the development of greater clonal heterogeneity at the time of diagnosis. Future work will be needed to validate these observations, via high-depth targeted sequencing and subsequent characterization of subclonal entities.

We also examined the host genome for germline variants that might be associated with IBC. Currently no genetic predisposing factors are known for IBC, but some familiar occurrences have been reported and IBC is more prevalent in certain geographic regions that suggest genetic contribution to its etiology [47, 48]. We identified heterozygous germline alterations in the TGFβ pathway that appear to be more frequent in IBC than in non-IBC (50% vs. 13%). Due to the rarity of IBC (0.5–2% of all breast cancers), our sample size is very small and this observation will need to be confirmed in larger independent IBC datasets. However, TGFβ has been implicated in the biology of IBC. *USP9X*, affected in 4 out of 20 IBC cases by a germline variant, is a deubiquitinating enzyme that controls *SMAD4* mono-ubiquitination and therefore affects TGFβ signaling [49]. One previous study showed that the expression of TGF *β* signaling pathway components are lower in IBC compared to non-IBC, and this may contribute to tumor emboli formation and facilitate lymphatic invasion of IBC cells [40]. Another study on head and neck cancer reported that loss of *SMAD4* was associated with increased TGF *β* 1 activity [50]. Overall, these results suggest the possibility of aberrant host TGF *β* signaling contributing to IBC biology and predisposition.

We recognize that our results are descriptive and hypothesis generating in terms of biological importance of the findings. However, it is clear from our analysis that there is no shared DNA level pathognomonic alteration in IBC. The sample size of our study is small; nevertheless, it is the largest study so far to examine the whole genome of IBC. Previous genomic analyses included only a few hundreds of genes that were sequenced using targeted sequencing platforms (Additional file 8: Table S7) [7–9]. We observed lower mutation frequencies in *PIK3CA* in our IBC cohort than previous ones, which may be due to sampling bias arising from the small sample size, as PIK3CA was more frequently mutated in hormone receptor-positive (HR+) cancers, and our study had a lower fraction of HR+ cases [51].

Besides various genetic features discussed in this study, it has been shown that IBC is significantly different with non-IBC in several non-genetic factors, including lower prevalence of parous women, higher oral contraceptive use, and higher frequency of regular alcohol consumption [52].

Overall, our results suggest that IBC falls within the continuum of breast cancer in terms of its molecular make up. Its particularly aggressive phenotype may result from unique co-occurrence of heterozygous host germline polymorphisms with subtle effects on TGF *β* signaling and somatic mutations that together enable rapid growth and expansion of a malignant cell clone.

## Conclusions

Here we present the first complete genomic landscape of IBC by whole-genome sequencing of tumor and their matched normal samples. Even though there was no unique, shared genomic alteration in IBCs, we identified several subtle but intriguing genomic differences between IBC and non-IBC which could potentially explain its etiology and result in new diagnostic markers, but will require validation in independent datasets in future studies.

## Supplementary Information

**Additional file 1: Table S1.** Characteristics of IBC and non-IBC patients.

**Additional file 2: Table S2.** Technical details for sequencing.

**Additional file 3: Table S3.** Tumor purity of IBC samples.

**Additional file 4: Fig. S1-S10.** Supplementary Fig. S1-S10.

**Additional file 5: Table S4.** Significant peaks with somatic CNVs.

**Additional file 6: Table S5.** Cancer genes located in significant peaks with somatic CNVs.

**Additional file 7: Table S6.** Germline SNVs in TGF *β* pathway.

**Additional file 8: Table S7.** Summary of previously published IBC studies.

## Data Availability

The datasets generated and analyzed during the current study have been submitted to the European Genome-Phenome Archive (EGA) under accession number EGAS00001004117 (https://wwwdev.ebi.ac.uk/ega/studies/EGAS00001004117) [53].
