# Supplementary material for: Whole-genome sequencing of phenotypically distinct inflammatory breast cancers reveals similar genomic alterations to non-inflammatory breast cancers
Source: Genome Med. 2021 Apr 26;13:70. doi: 10.1186/s13073-021-00879-x (PMC8077918; doi:10.1186/s13073-021-00879-x)
Supplement: Supplementary file 4 — Additional file 4: Fig. S1-S10. Supplementary Fig. S1-S10. [file 13073_2021_879_MOESM4_ESM.pdf]

Fig S1

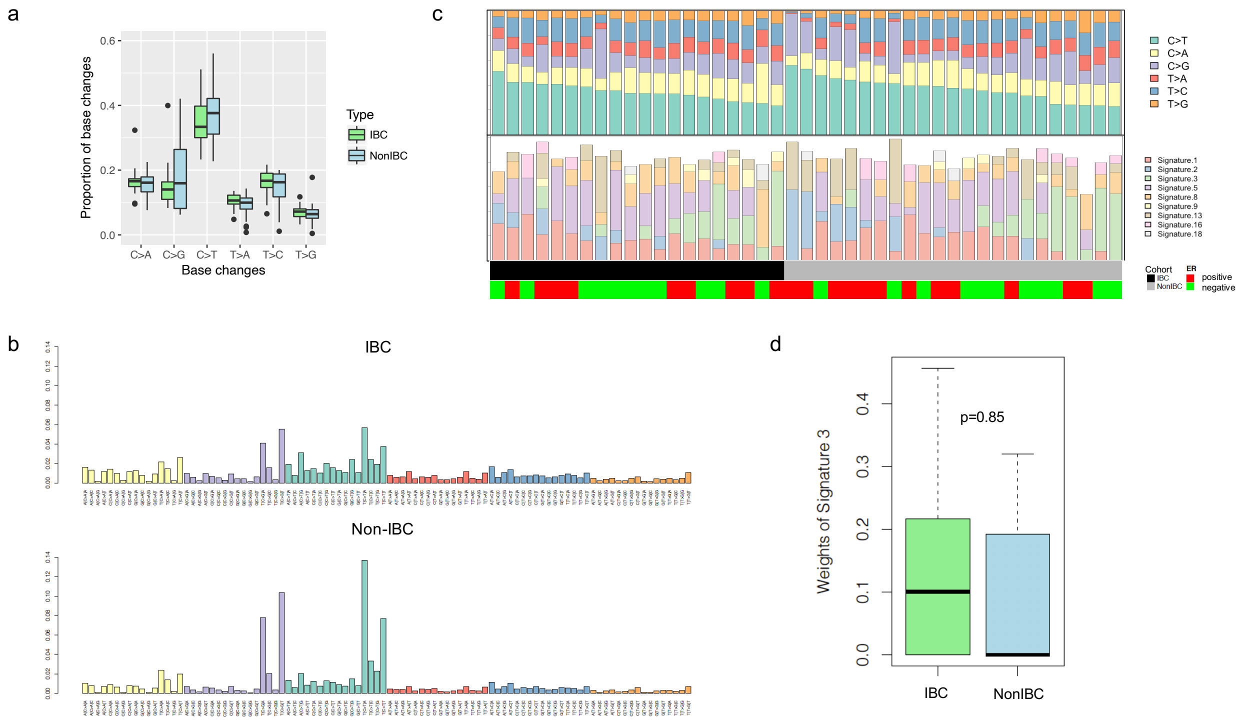

Fig S1. Mutation spectra and mutational signatures

a. Proportion of somatic base changes in IBC and non-IBC cohorts. **b.** Mutation spectra of IBC and non-IBC cohorts. **c.** Proportion of somatic base changes and weights of nine reference mutational signatures, for individual IBC and non-IBC cases, annotated with ER status. **d.** Comparison of weights of signature 3, which is associated with homologous recombination defect, in IBC and non-IBC cohorts. For panel a (base change), c and d (weights of signatures), two cohorts were compared by Wilcoxon rank sum test. Subsequent p-values were adjusted by Bonferroni method. All adjusted p-values>0.05.

Fig S2

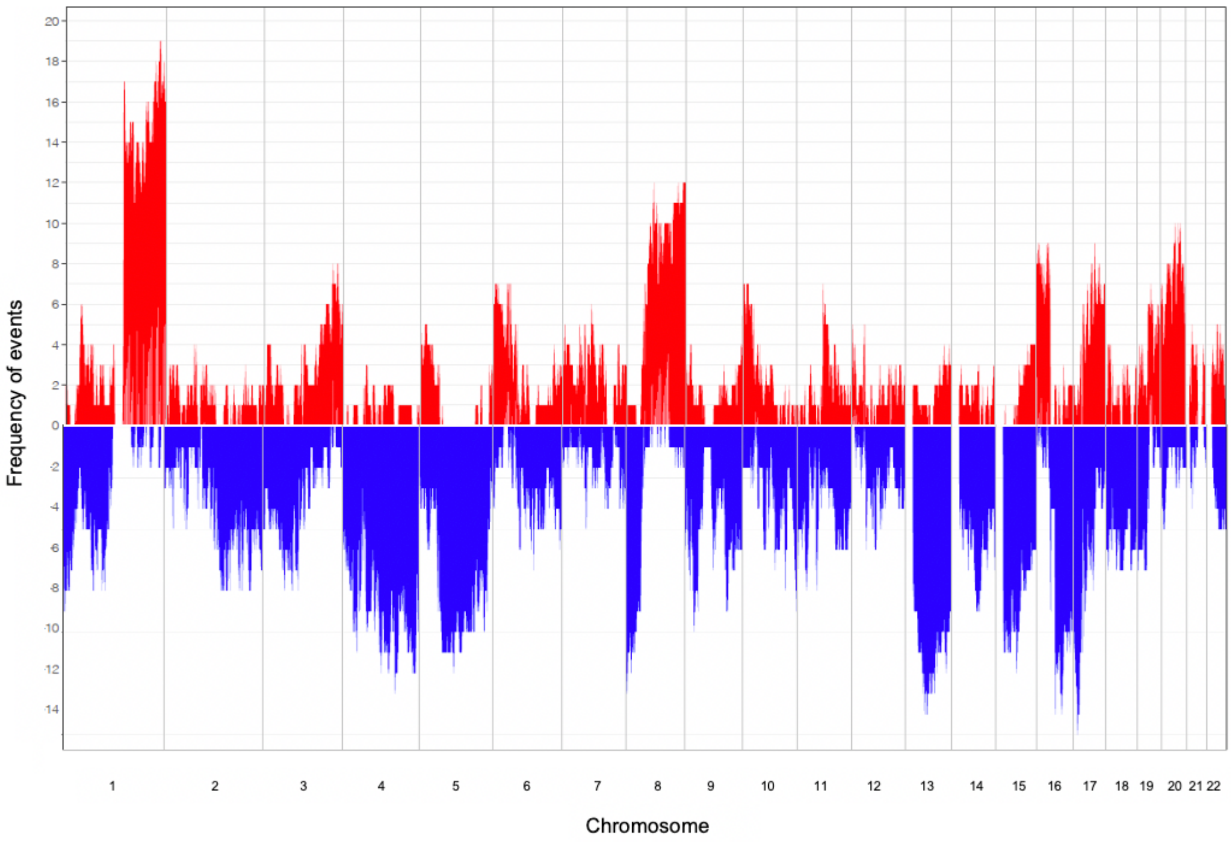

Fig S2. Somatic copy number profile of the non-IBC cohort

X-axis represents genome coordinates ordered by chromosomes. Y-axis shows the frequency of copy number gain (red) and copy number loss (blue) in 1Mb-length bins across the genome in non-IBCs.

Fig S3

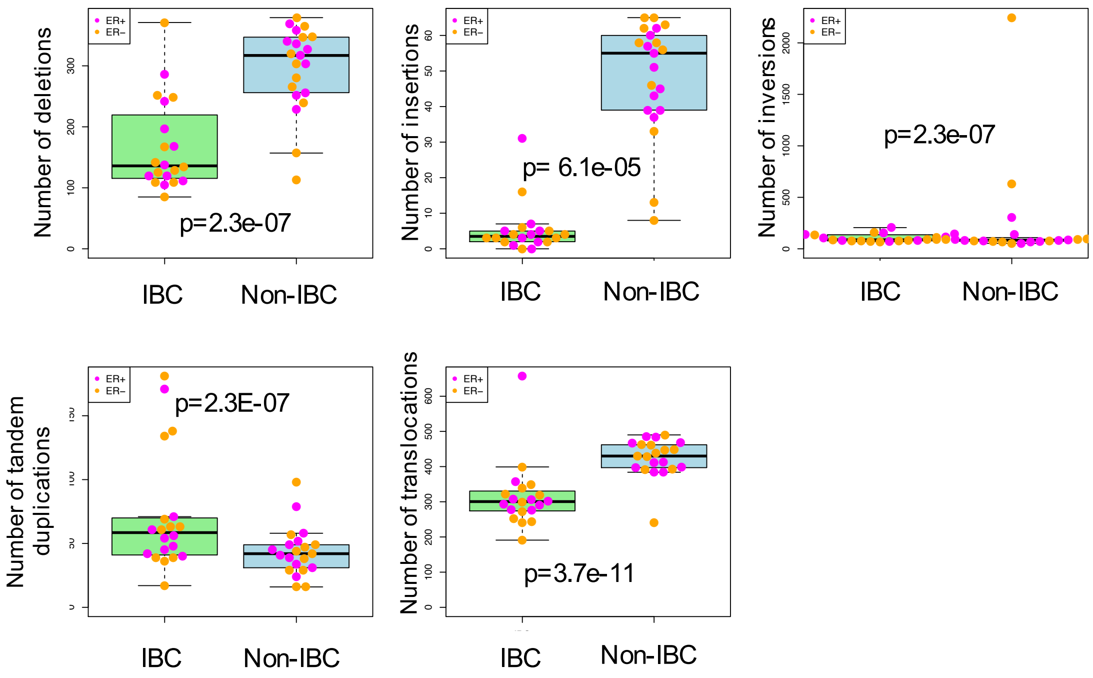

Fig S3. Number of somatic structural variants in IBC and non-IBC cohorts

Number of somatic SVs in each category in IBC and non-IBC cohorts. Each dot represents a sample color-coded by its ER status. P-values were calculated by Wilcoxon test and adjusted by Bonferroni method.

Fig S4

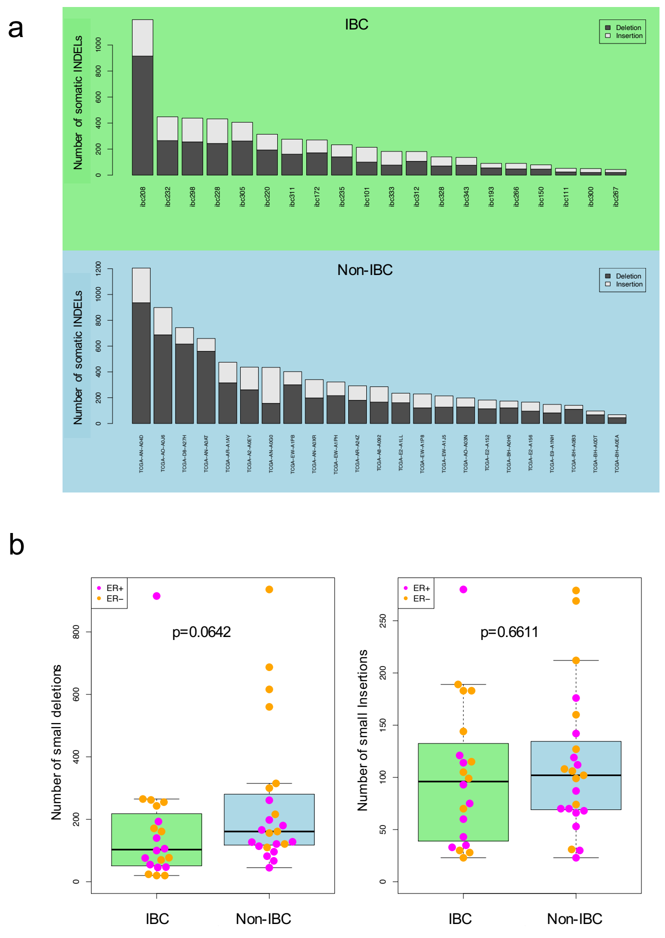

Fig S4. Number of small insertions and deletions (INDELs) in IBC and non-IBC cohorts

a. Number of small INDELs in individual IBC and non-IBC samples. Shades represent the types of small INDELs. **d.** Number of small deletions and small insertions in IBC and non-IBC cohorts. Each dot represents a sample color-coded by its ER status. P-values were calculated by Wilcoxon test.

Fig S5

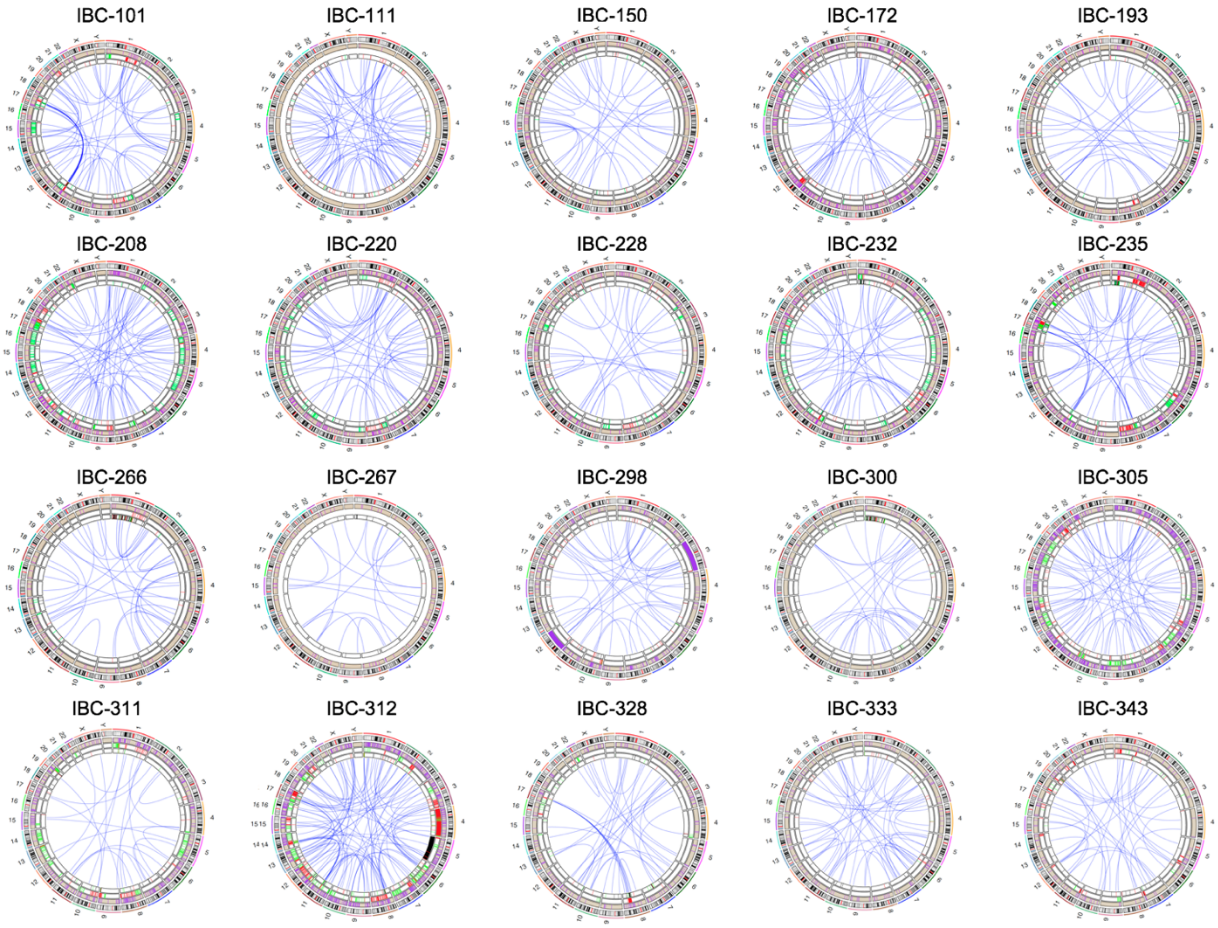

Fig S5. Circos plots of individual IBC cases

The outer ring represents each chromosome ideogram, the next ring is the somatic SNV (purple), the next ring is somatic CNV (red: gain; green: loss), the inner ring is somatic SV (red: tandem duplication; green: deletion; black: inversion). The interchromosomal translocations are shown by the lines in the center of the plot.

Fig S6

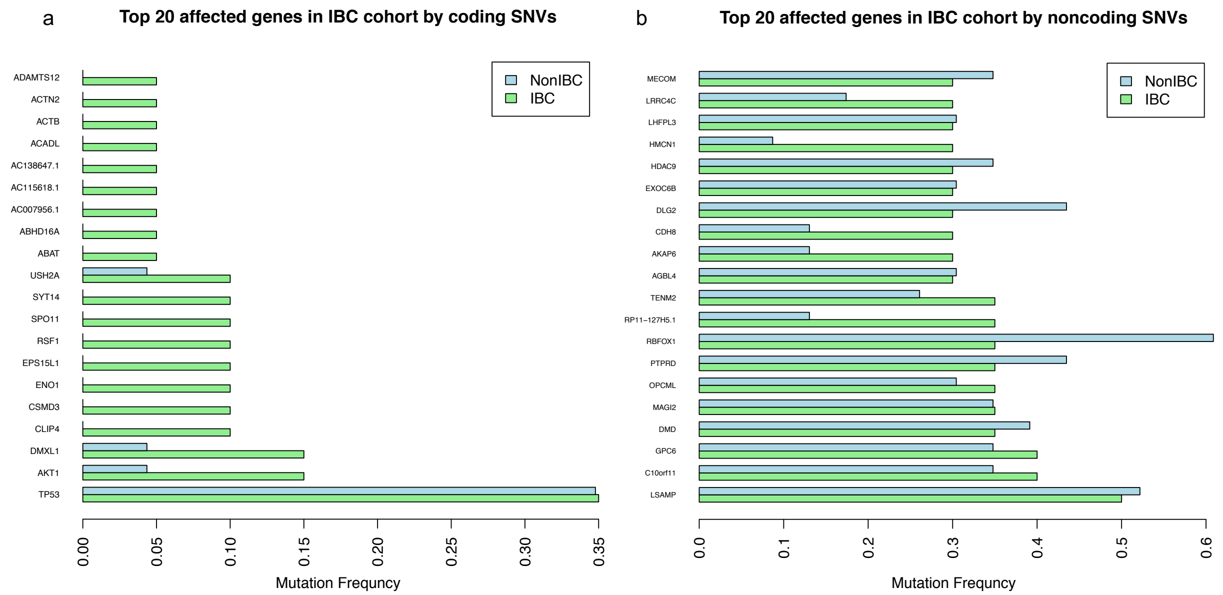

Fig S6. Top 20 most frequently affected genes by coding and non-coding SNVs

a. Top 20 most frequently affected genes by coding SNVs in IBC. Green and blue bars represented mutation frequency of a given gene in IBC and non-IBC cohort, respectively. **b.** Top 20 most frequently affected genes by noncoding SNVs in IBC. Green and blue bars represented mutation frequency of a given gene in IBC and non-IBC cohort, respectively. For all genes in **a** and **b**, their mutation frequencies were not significantly different between IBC and non-IBC cohorts (Adjusted p-values>0.05, Fisher's exact test, Bonferroni correction).

Fig S7

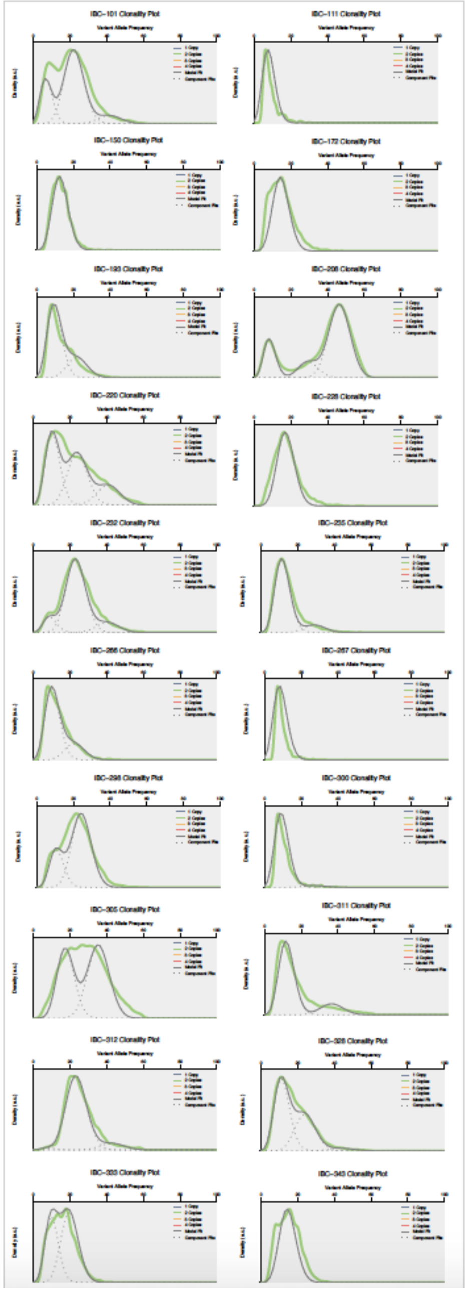

Fig S7. Clonality Plot for each individual IBC sample

Clonality plot presented the distribution of variant allele frequencies for all input somatic SNVs for each sample (green line), as well as the model fit (grey solid line) and the component fits (grey dashed line) results from the SciClone method.

Fig S8

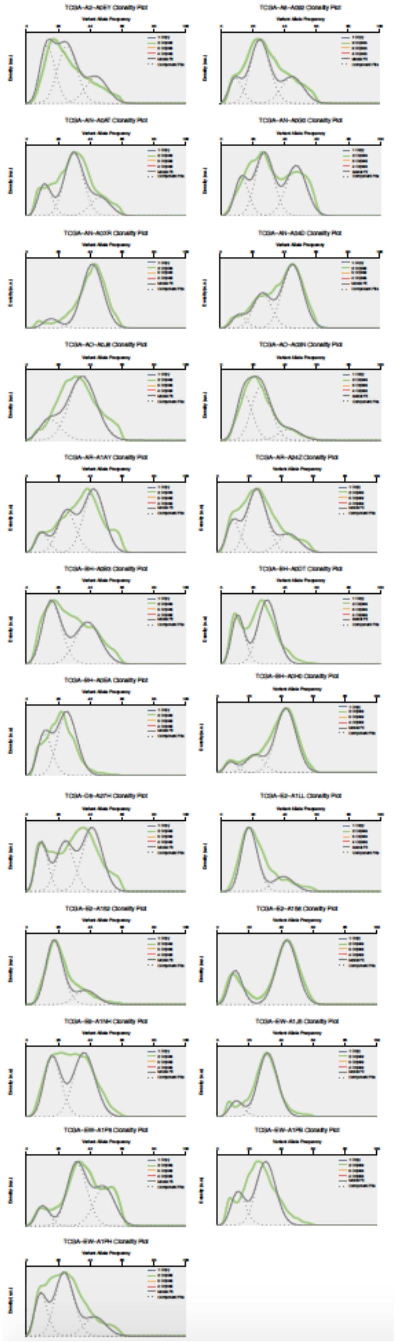

Fig S8. Clonality Plot for each individual non-IBC sample

Clonality plot presented the distribution of variant allele frequencies for all input somatic SNVs for each sample (green line), as well as the model fit (grey solid line) and the component fits (grey dashed line) results from the SciClone method.

Fig S9

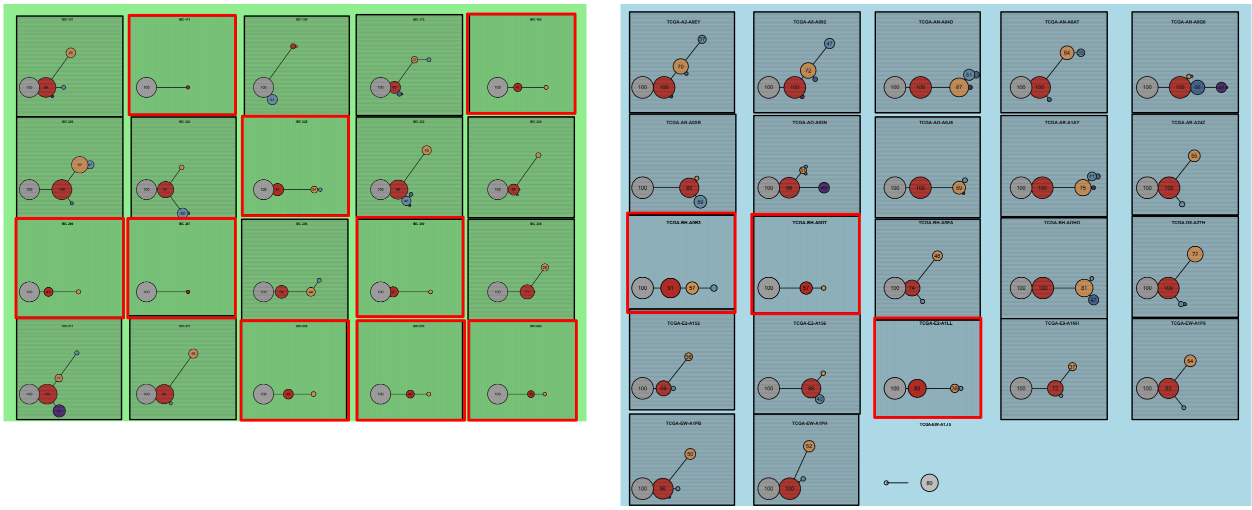

Fig S9. Individual evolutionary trees of IBC and non-IBC cohorts

Each sample is shaded with horizontal or vertical lines to indicate branching and linear pattern, respectively. Samples belonging to the linear group are also highlighted by red.

Fig S10

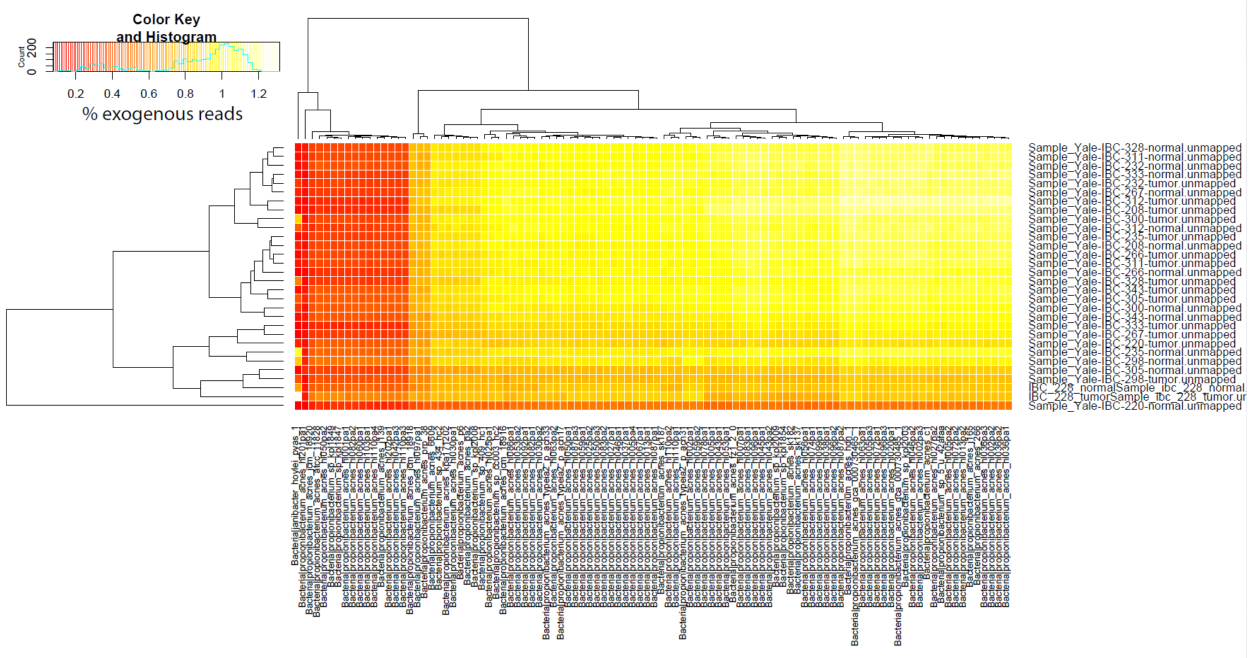

Fig S10. Top 100 most frequent non-human sequences in the IBC cancer and normal DNA

Each row is one IBC cancer or normal sample. Each column is one microorganism. Color scales correspond to the percentage of exogenous reads detected in a given sample, for the indicated microorganism.
